# Effects of different intensities of continuous training on vascular inflammation and oxidative stress in spontaneously hypertensive rats

**DOI:** 10.1111/jcmm.16813

**Published:** 2021-07-31

**Authors:** Minghao Luo, Chunmei Cao, Josef Niebauer, Jianghong Yan, Xindong Ma, Qing Chang, Ting Zhang, Xiaoxiao Huang, Guochun Liu

**Affiliations:** ^1^ Department of Cardiology The First Affiliated Hospital of Chongqing Medical University Chongqing China; ^2^ Division of Sports Science and Physical Education Tsinghua University Beijing China; ^3^ University Institute of Sports Medicine, Prevention and Rehabilitation Paracelsus Medical University Salzburg Austria; ^4^ Institute of Life Sciences Chongqing Medical University Chongqing China; ^5^ The College of Exercise Medicine Chongqing Medical University Chongqing China; ^6^ The Fifth Affiliated Hospital of Sun Yat‐sen University Guangdong China

**Keywords:** blood vessel, oxidative stress, spontaneously hypertensive rats, training intensity, vascular inflammation

## Abstract

We aimed to study the effects and underlying mechanism of different intensities of continuous training (CT) on vascular inflammation and oxidative stress in spontaneously hypertensive rats (SHR). Rats were divided into five groups (*n* = 12): Wistar‐Kyoto rats sedentary group (WKY‐S), sedentary group (SHR‐S), low‐intensity CT group (SHR‐L), medium‐intensity CT group (SHR‐M) and high‐intensity CT group (SHR‐H). Changes in body mass, heart rate and blood pressure were recorded. The rats were euthanized after 14 weeks, and blood and vascular tissue samples were collected. Haematoxylin and Eosin staining was used to observe the aortic morphology, and Western blot was used to detect the expression of mesenteric artery proteins. After CT, the mean arterial pressures improved in SHR‐L and SHR‐M and increased in SHR‐H compared with those in SHR‐S. Vascular inflammation and oxidative stress levels significantly subsided in SHR‐L and SHR‐M (*p *< 0.05), whereas in SHR‐H, only vascular inflammation significantly subsided (*p *< 0.05), and oxidative stress remained unchanged (*p *> 0.05). AMPK and SIRT1/3 expressions in SHR‐L and SHR‐M were significantly up‐regulated than those in SHR‐S (*p *< 0.05). These results indicated that low‐ and medium‐intensity CT can effectively reduce the inflammatory response and oxidative stress of SHR vascular tissue, and high‐intensity CT can improve vascular tissue inflammation but not oxidative stress.

## 1 INTRODUCTION

Hypertension is a complex disease and a risk factor for other cardiovascular diseases. Vascular dysfunction is considered an important indicator of the development of hypertension.^1^ There is sufficient evidence that cardiovascular disease is related to increased inflammation and oxidative stress.^2^, ^3^ Toll‐like receptor 4 (TLR4),^4^ nuclear factor kappa B (NF‐κB)^5^, ^6^, ^7^ and NOD‐, LRR‐ and pyrin domain‐containing protein 3 (NLRP3)^8^, ^9^, ^10^ are directly involved in the increased inflammation and oxidative stress associated with the hypertensive cardiovascular system. Balancing vascular redox reactions in hypertension can protect cardiovascular homeostasis.^1^

Toll‐like receptors (TLRs) are key members of cell transmembrane receptors and pathogenic membrane recognition receptors in the innate immune system. TLR4 contributes to inflammation and oxidative stress and is associated with endothelial dysfunction and vascular remodelling in hypertension.^4^ TLRs promote the release of inflammatory cytokines by recognizing aggressive immune response pathogens and interacting with NF‐κB. Phosphorylation of IκB and p65 induces the degradation of IκB and subsequent translocation of p65 into the nucleus, thus activating the NF‐κB pathway, which further synthesizes pro‐inflammatory cytokines.^5^ For example, tumour necrosis factor‐α (TNF‐α), interleukin‐6 (IL‐6) and interleukin‐1β (IL‐1β) cause chronic low‐grade inflammation in hypertension. Additionally, NLRP3 inflammasome can promote the conversion of IL‐18 and IL‐1β precursors into mature IL‐18 and IL‐1β, which plays a key role in atherosclerosis.^9^ Increasing studies have confirmed that NLRP3 inflammasome is involved in the occurrence and development of hypertension.^10^

In addition to the role of inflammation, free radicals, such as reactive oxygen species (ROS) and reactive nitrogen species (RNS), are involved in the pathological progress of hypertension.^11^ More importantly, inflammatory factors can activate ROS,^12^ which in turn activate a variety of intracellular signal transduction pathways, including NF‐κB and NLRP3, leading to a further increase in ROS production; thus, a positive feedback mechanism is formed, which ultimately leads to the progression of hypertension.^12^ ROS derived from NADPH oxidase (NOX) is an important signalling molecule in endothelial cells (ECs) and vascular smooth muscle cells (VSMCs), and it is involved in cell growth, migration, inflammation, fibrosis and contraction.^13^ In hypertension, the increased activity of the three subtypes of NOX (NOX1, NOX2 and NOX4) in blood vessels is related to oxidative stress and abnormal redox signals, leading to ECs and VSMCs dysfunction, which further causes vascular damage.^14^ Superoxide dismutase 2 (SOD2) is an antioxidant enzyme that can catalyse the disproportionation of superoxide anion radicals to oxygen and hydrogen peroxide and plays a crucial role in the balance of oxidation and antioxidants in vivo.^15^

At present, continuous training (CT) is an effective strategy for the treatment of hypertension, and its induction of adaptive changes in the blood vessel wall (including ECs and VSMCs) has been supported by experimental and clinical studies.^16^, ^17^, ^18^, ^19^ In the early studies, CT was shown to affect arterial blood vessels mainly through improving risk factors, such as blood pressure, blood lipid levels, insulin resistance and obesity to indirectly affect the function and morphology of arterial blood vessels.^20^, ^21^ However, more studies later showed that CT directly provides a wide range of benefits to alleviate hypertension. For example, it can promote angiogenesis,^22^ improve vascular structure,^19^ improve inflammation^23^ and balance oxidative stress.^24^, ^25^, ^26^ Studies have shown that CT increases the production and bioavailability of nitric oxide, which is mediated by a variety of pathways, including the synthesis of molecular mediators, changes in neurohormone release and oxidant/antioxidant balance. Furthermore, CT can affect systemic molecular pathways related to angiogenesis and chronic anti‐inflammatory effects, thereby affecting vascular function and structural changes.^27^ In research involving CT and hypertension, the effects of different training intensities have increasingly been studied; different training intensities include physical activity, low‐intensity training, medium‐intensity training, high‐intensity interval training. Changes in vascular structure and function may be related to the intensity of training load^22^, ^23^, ^28^; therefore, investigating the effect of training intensity in hypertensive exercise rehabilitation is particularly important.

Therefore, we hypothesized that CT might improve blood pressure by altering oxidant and inflammatory profiles in the vascular tissue of spontaneously hypertensive rats (SHR). This study aims to explore the effects of different intensities of CT on vascular inflammation (TLR4/NF‐κB/NLRP3) and oxidative stress (SOD2, NOX2/4) in SHR and investigate the associated underlying mechanism of action. This study reported the role of training in regulating NF‐κB and NLRP3 pathways in SHR for the first time.

## 2 MATERIALS AND METHODS

### 2.1 Animals and CT protocol

All research procedures were approved by the Ethics Committee of Chongqing Medical University. The experiment complied with the regulations of the People's Republic of China's ‘Regulations on the Management of Laboratory Animals (2017 Revision)’ and the requirements of the Guidelines for Animal Exercise and Training Protocols for Cardiovascular Studies. Eight‐week‐old male SHR were divided into four groups (*n* = 12): sedentary (SHR‐S); low‐intensity continuous training (LICT) (SHR‐L), with a running speed of 14 m/min and approximately 35% of maximum oxygen uptake (VO_2_ max); medium‐intensity continuous training (MICT) (SHR‐M), with a speed of 20 m/min and approximately 50% VO_2_ max; and high‐intensity continuous training (HICT) (SHR‐H), with a speed of 26 m/min and approximately 65% VO_2_max.^29^ Age‐matched male Wistar‐Kyoto rats were regarded as a sedentary control (WKY‐S). The experimental rats were provided by Vital River Laboratory Animal Technology Co. Ltd., weighing about 190–200 g.

The rats were kept under uniform conditions in the Experimental Animal Center of Chongqing Medical University: 5 rats in each cage, approximately 22℃ room temperature, a regular light cycle (12:12 h of light and dark), humidity maintained at 40%–45%, tap water and feed freely accessible, and all experimental environments reached specific pathogen‐free (SPF). The rats were trained on a treadmill (SA101C, Sans) for 14 weeks, 5 times a week and 60 min each time. The treadmill inclination of each training group was 0. Chocolate (0.5 g) was given as a reward after each training session. After the experiment, the rats were euthanized. The aorta was excised to assess vascular function and morphometry, and the mesenteric artery was used for Western blot analysis.

### 2.2 Materials and drugs

Primary antibodies against eNOS (1:2000), p‐eNOS (Ser1177) (1:1000), iNOS (1:2000), p65 (1:2000), Phospho‐p65 (Ser536) (1:1000), IκBα (1:2000), Phospho‐IκBα (Ser32) (1:1000), AMPKα (1:2000), Phospho‐AMPKα (Thr172) (1:1000), SIRT1 (1:2000) and SIRT3 (1:2000) were purchased from Cell Signaling Technology. Primary antibodies against NLRP3 (1:1000), Caspase‐1 (1:1000), ASC (1:1000), IL‐1β (1:1000), and 3‐nitrotyrosine (3‐NT) (1:1000) were obtained from Bioss (Beijing, China). Primary antibodies against TLR4 (1:2000), SOD2 (1:2000), NOX2 (1:2000), NOX4 (1:2000), GAPDH (1:5000) and secondary antibodies (goat‐anti‐rabbit) (1:5000) were obtained from Proteintech Group. Unless otherwise indicated, all other chemicals used in this study were obtained from Sigma‐Aldrich.

### 2.3 Weight, heart rate and blood pressure measurements

The rats were weighed using an animal scale (UX/UW, Shimadzu) from 8 to 12 a.m. every Monday. Systolic blood pressure (SBP), diastolic blood pressure (DBP), mean arterial pressure (MAP) and heart rate (HR) were measured in conscious rats using a non‐invasive tail‐cuff system (Softron). Rats were habituated to the tail‐cuff procedure prior to experimentation. Before the measurements, the rats were kept in an incubator (37℃) for 10 min. The blood pressure of each rat was obtained by averaging three measurements.

### 2.4 Vascular morphometry analysis

A 1.5‐cm section of the ascending thoracic aorta was dissected from each rat. Paraffin sections (5 μm) were cut and stained with haematoxylin and eosin. ImageJ 1.52v software was used to calculate the thickness and cross‐sectional area of the vascular intima‐media layer. Mean value of the vessel wall thickness from the endothelial surface to the boundary between medial and adventitia of blood vessel was determined using five different locations spanning the entire cross‐section. All images were captured by a Leica DM4B upright metallurgical microscope (Leica Inc).

### 2.5 Western blot analysis

Protein expression was measured by Western blot.^30^, ^31^ Briefly, tissues were lysed on ice for 1 h using a lysis buffer. The protein concentration was measured using the Bradford assay (Beyotime), and 40 μg of protein was used for the Western blot. Proteins were separated using 8%–10% SDS‐PAGE and transferred onto PVDF membranes; the membranes were blocked with 5% non‐fat milk for 1 h at 22℃ and then incubated with the primary antibodies overnight at 4℃. Membranes were reacted with secondary antibodies conjugated with horseradish peroxidase at 22℃ for 1 h. Bands were detected by chemiluminescence detection reagent (Beyotime). The grey value of the bands was analysed using Image Lab 6.0, and the level of the target protein was represented by the ratio of the grey value of the target protein to the internal control protein (GAPDH). The level of phosphorylation was indicated by the ratio of the grey value of the phosphorylated protein to the total protein.

### 2.6 Vascular reactivity experiment

The thoracic aorta was immediately dissected and put in physiological salt solution (PSS) buffer (mmol/L): glucose 5.5, NaCl 119, NaHCO_3_ 25, KCl 4.7, CaCl_2_ 2.5, KH_2_PO_4_ 1.2 and MgSO_4_ 1.2.^31^ Adhered tissues were removed, and aorta was chopped into 3‐mm rings and moved into the Multi Myograph System chambers (DMT). The rings were mounted on chambers filled with warmed (37℃), oxygenated (air mixture containing 95% O_2_ and 5% CO_2_) PSS. LabChart software (DMT620) was used to continuously record the vascular tension. The aortic rings were maintained at a basal tension of 2 g for 90 min and then stimulated twice with KCl (60 mmol/L)‐PSS in advance. Relaxation of norepinephrine (NE, 10^−7^ M) precontracted vessels to acetylcholine (ACh, 10^−5^ M) was used to determine endothelial integrity (vessels that relaxed by at least 80% were deemed endothelium intact). Following this, aortas were then precontracted with NE (10^−7^ M), until the aortic rings reached maximum contraction and the tension curve became stable, cumulative concentration‐response (10^−9^ M to 10^−5^ M) curves were constructed for ACh (endothelium‐dependent relaxation). Average concentration‐response curves were plotted.

### 2.7 Analysis of biochemical parameters

The antioxidant and oxidative stress indicators in serum were measured using reagent kits provided by the Nanjing Jiancheng Bioengineering Institute (Nanjing, China). SOD was tested using the hydroxylamine method; glutathione peroxidase (GSH‐Px) was tested using the colorimetric method; malondialdehyde (MDA) was tested using the thiobarbituric method (TBA method).

### 2.8 Statistical analysis

All data were expressed as mean ± SEM. Statistical evaluation was performed using one‐way or two‐way ANOVA, followed by post hoc tests with Bonferroni adjustments. Statistical significance was set at *p *< 0.05. The GraphPad Prism 8.0 was used for this purpose.

## 3 RESULTS

### 3.1 Basic data (effects of different training intensities on the body mass, blood pressure and heart rate of rats)

**FIGURE 1:**
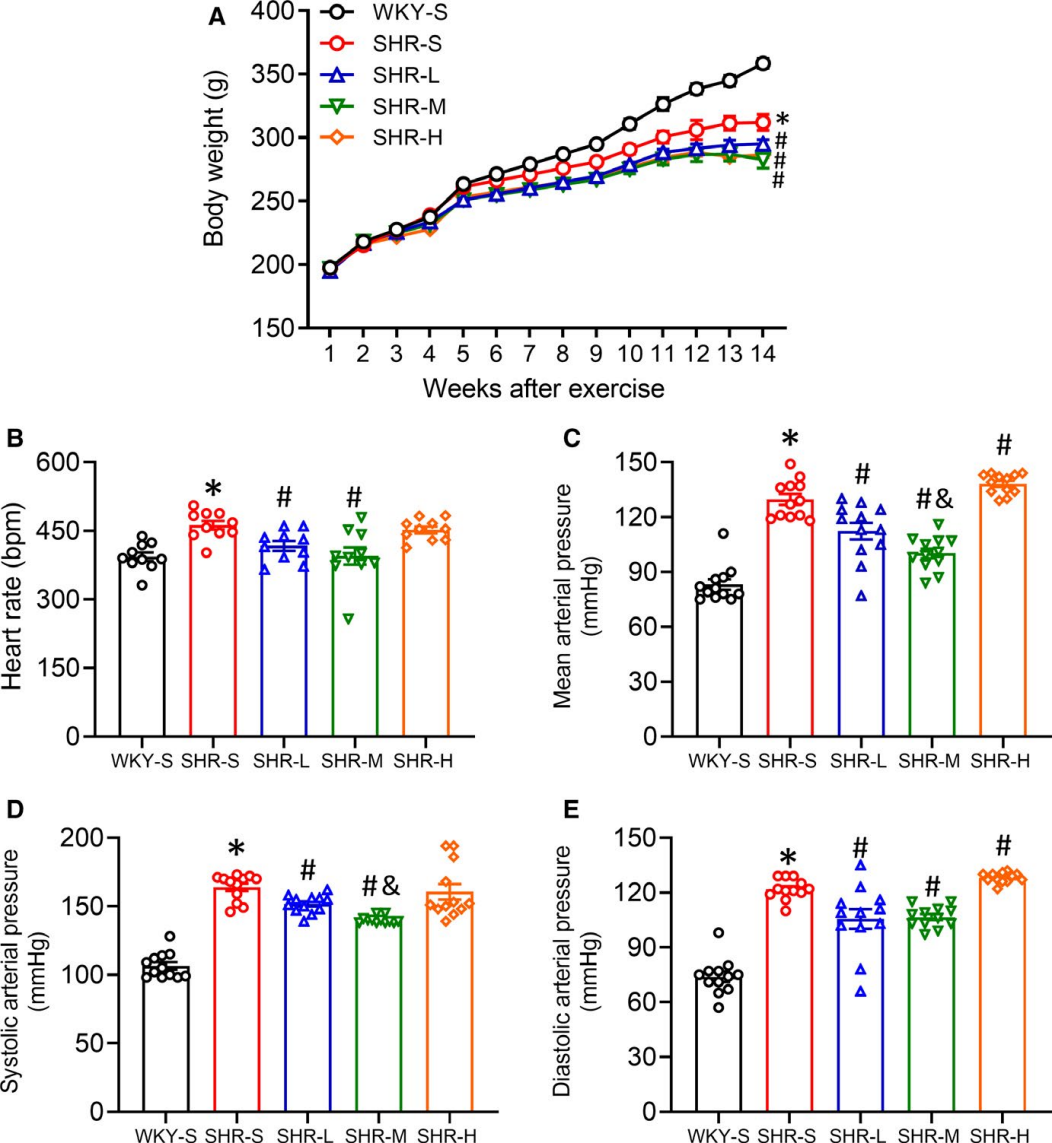
Effects of different‐intensity continuous training on body mass, heart rate and blood pressure in SHR. Wistar‐Kyoto rats sedentary group (WKY‐S), spontaneously hypertensive rats sedentary group (SHR‐S), SHR LICT group (SHR‐L), SHR MICT group (SHR‐M) and SHR HICT group (SHR‐H) were trained on the treadmill with different intensities for 14 weeks, changes of body mass were recorded (A). Heart rate (B), mean arterial pressure (C), systolic blood pressure (D) and diastolic blood pressure (E) were measured with the tail‐cuff method after training. *p < 0.05 compared with WKY‐S, #*p *< 0.05 compared with SHR‐S, ^&^ *p *< 0.05 compared with SHR‐L

We measured the weight of the rats once a week and observed different degrees of increase in the weight of each group (Figure 1A). There was a significant difference in weight between WKY‐S and SHR‐S (WKY‐S 358.5 and SHR‐S 311.9 at the 14^th^ week; *p *< 0.05) and none among SHR‐L, SHR‐M and SHR‐H. Additionally, significant differences in weight were observed between the training groups and SHR‐S (SHR‐L 295.0, SHR‐M 282.7, SHR‐H 286.5 at the 14^th^ week; *p *< 0.05). We used the tail‐cuff method to detect heart rate and blood pressure at the 14^th^ week (Figure 1B–E). The HR of SHR‐S was higher than that of WKY‐S (WKY‐S 377.3 and SHR‐S 461.8; *p *< 0.05). There were significant differences in the HR of SHR‐L and SHR‐M compared with that of SHR‐S (SHR‐L 408.8 and SHR‐M 388.2; *p *< 0.05). SBP, DBP and MAP of SHR increased significantly (SBP: WKY‐S 106.5, SHR‐S 163.8; DBP: WKY‐S 73.9, SHR‐S 121.9; MBP: WKY‐S 83.2, SHR‐S 130.0; *p *< 0.05). LICT and MICT significantly reduced the SBP, DBP and MAP of SHR (SBP: SHR‐L 151.8, SHR‐M 140.1; DBP: SHR‐L 105.6, SHR‐M 106.6; MBP: SHR‐L 112.3, SHR‐M 100.2, *p *< 0.05), of which MICT had a better effect (*p *< 0.05). The DBP and MAP of the SHR‐H were significantly higher than those of SHR‐S (DBP: SHR‐H 128.1; MBP: SHR‐H 138.2; *p *< 0.05).

### 3.2 Vascular structure

**FIGURE 2:**
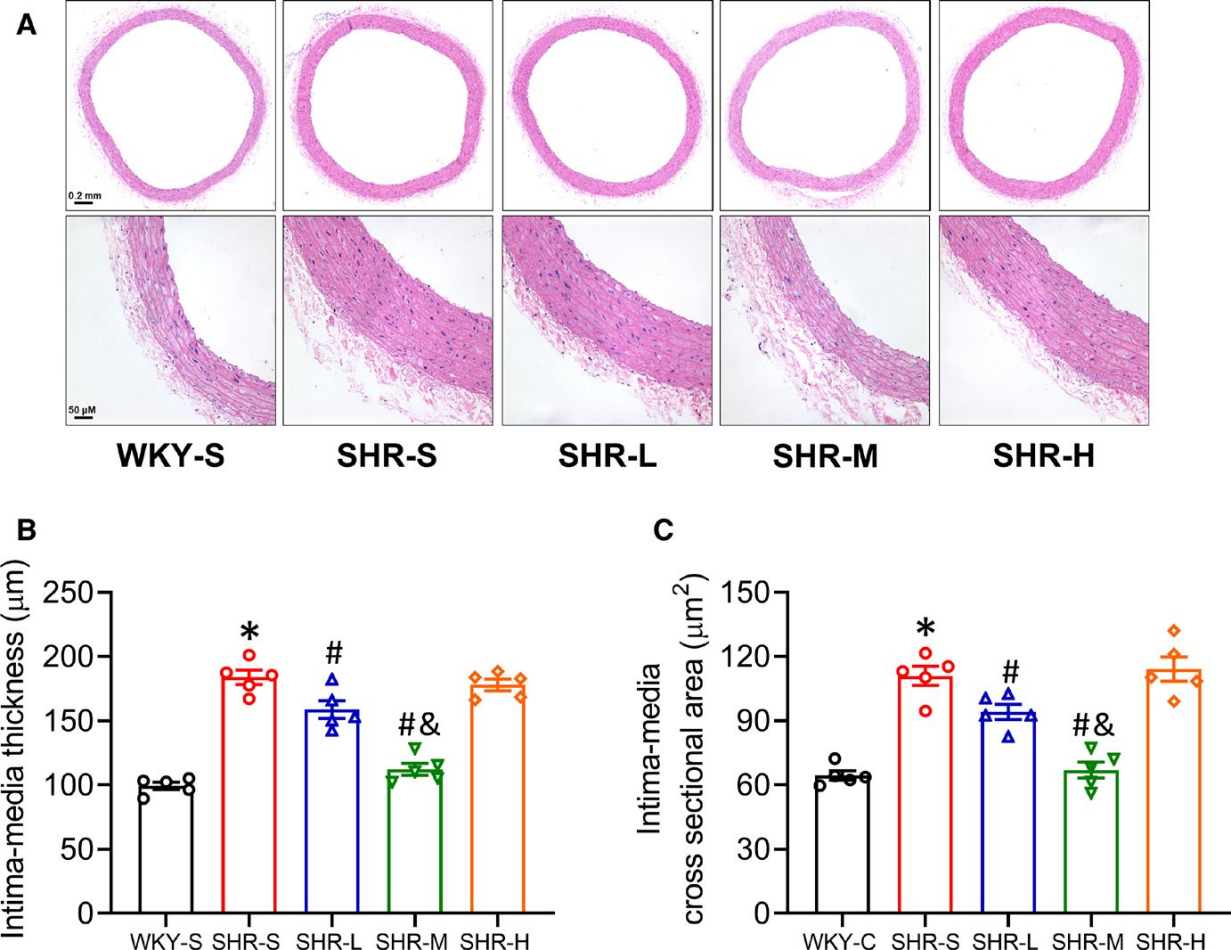
Effects of different‐intensity continuous training on vascular structure in SHR. WKY‐S, SHR‐S, SHR‐L, SHR‐M and SHR‐H were trained on the treadmill with different intensities for 14 weeks. Representative haematoxylin and eosin‐stained images of the aorta in each group (original magnification ×50 and ×200, respectively) were presented (A). The intima‐media thickness (B) and cross‐sectional area (C) values were quantified using ImageJ software. *p < 0.05 compared with WKY‐S, #*p *< 0.05 compared with SHR‐S, &*p *< 0.05 compared with SHR‐L

To explore the potential impact of different intensities of training on the vascular morphology of SHR, the vascular morphology of the thoracic aortas of the rats involved in this study was examined using slice staining technology (Figure 2A). Using ImageJ to calculate the thickness and cross‐sectional area of the vascular intima‐media layer, we observed that SHR‐S arterial intima‐media was significantly thicker than that of WKY‐S (WKY‐S 99.3, SHR‐S 183.8; *p *< 0.05) and thinner in SHR‐L and SHR‐M than SHR‐S (SHR‐L 158.8, SHR‐M 112.3, *p *< 0.05), of which MICT had a better effect (*p *< 0.05). SHR‐H did not change in thickness of the vascular intima‐media layer compared with SHR‐S (SHR‐H 178.0, *p *> 0.05). The cross‐sectional area of the intima‐media in SHR‐S was significantly larger than that in WKY‐S (WKY‐S 64.4 and SHR‐S 110.9; *p *< 0.05), and there was a significant decrease in the cross‐sectional area of intima‐media in SHR‐L and SHR‐M compared with that in SHR‐S (SHR‐L 94.2 and SHR‐M 67.0; *p *< 0.05), whereas there was no statistical difference in the cross‐sectional area of intima‐media between SHR‐H and SHR‐S (SHR‐H 114.2; *p *> 0.05).

### 3.3 Changes of p‐eNOS, eNOS, iNOS and 3‐NT expression in the mesenteric artery

**FIGURE 3:**
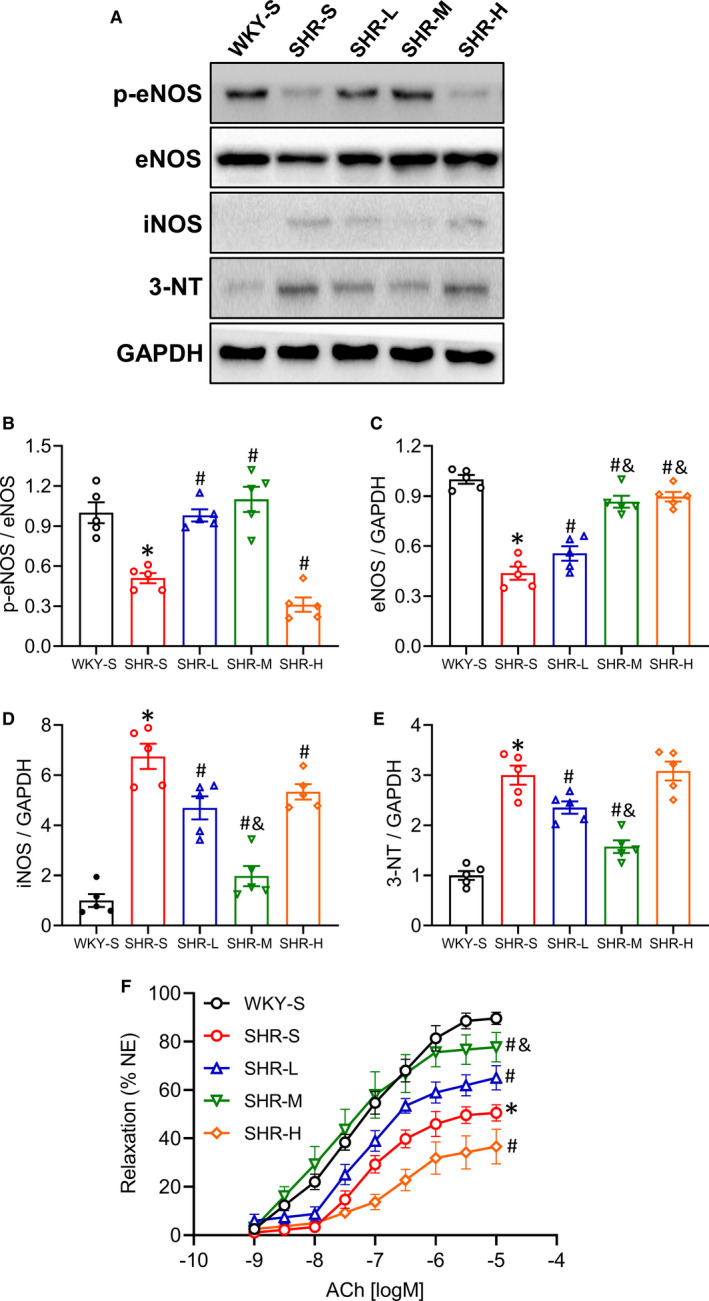
Effects of different‐intensity continuous training on p‐eNOS, eNOS, iNOS, 3‐NT expression and relaxation function in SHR. WKY‐S, SHR‐S, SHR‐L, SHR‐M and SHR‐H were trained on the treadmill with different intensities for 14 weeks. After training, the protein expression of p‐eNOS (B), eNOS (C), iNOS (D), 3‐NT (E) in mesenteric artery were determined by Western blot (A), acetylcholine (ACh)‐induced relaxation of aorta was measured by vascular reactivity experiment (F). *p < 0.05 compared with WKY‐S, #*p *< 0.05 compared with SHR‐S, &*p *< 0.05 compared with SHR‐L

Nitric oxide (NO) plays an important role in the regulation of vascular function. In the cardiovascular system, endothelial nitric oxide synthase (eNOS) and inducible nitric oxide synthase (iNOS) mainly produce NO and eNOS phosphorylation site at serine 1177 plays an essential role in the production of NO in vascular endothelial cells. Besides, 3‐nitrotyrosine (3‐NT) is the most commonly used peroxynitrite biomarker in biological systems. We used Western blot to detect the expression of p‐eNOS(Ser1177), eNOS, iNOS and 3‐NT in the mesenteric artery of rats (Figure 3A). The expression of p‐eNOS (Figure 3B) and eNOS (Figure 3C) in the mesenteric artery in SHR‐S decreased than that in WKY‐S (*p *< 0.05), and the expression of iNOS (Figure 3D) and 3‐NT (Figure 3E) increased (*p *< 0.05). Compared with SHR‐S, p‐eNOS and eNOS expressions increased (*p *< 0.05), and iNOS and 3‐NT expressions decreased in SHR‐L and SHR‐M (*p *< 0.05). In the SHR‐H, the p‐eNOS expression decreased but there were no significant differences in 3‐NT expression than that in SHR‐S. (p‐eNOS: SHR‐S 0.51, SHR‐L 0.98, SHR‐M 1.10 and SHR‐H 0.31‐fold compared with WKY‐S; eNOS: SHR‐S 0.44, SHR‐L 0.55, SHR‐M 0.87, SHR‐H 0.89‐fold compared with WKY‐S; iNOS: SHR‐S 6.55, SHR‐L 4.70, SHR‐M 1.97, SHR‐H 5.34‐fold compared with WKY‐S; 3‐NT: SHR‐S 3.00, SHR‐L 2.36, SHR‐M 1.57, SHR‐H 3.08‐fold compared with WKY‐S).

### 3.4 Change of vasodilation function of aorta

Decreased eNOS activity in vascular endothelial cells is a hallmark of endothelial dysfunction, characterized by impaired endothelium‐dependent relaxation (ACh‐induced), which is an early marker for hypertension. We explored the changes in vasodilation function of different groups. Results were analysed by using two‐way ANOVA. Compared with WKY‐S, ACh‐induced dilation function was impaired in SHR‐S (*p *< 0.05) (Figure 3F). LICT and MICT significantly improved ACh‐induced vasodilation function of aorta, compared with SHR‐S (*p *< 0.05). However, HICT impaired the ACh‐induced vasodilation function in SHR (*p* < 0.05). These results indicate that LICT and MICT have a protective effect on the endothelium‐dependent relaxation function in SHR, whereas HCT has a potentially harmful effect on the endothelium‐dependent relaxation function.

### 3.5 Changes in TLR4, p‐p65 and p‐IκBα expression in the mesenteric artery

**FIGURE 4:**
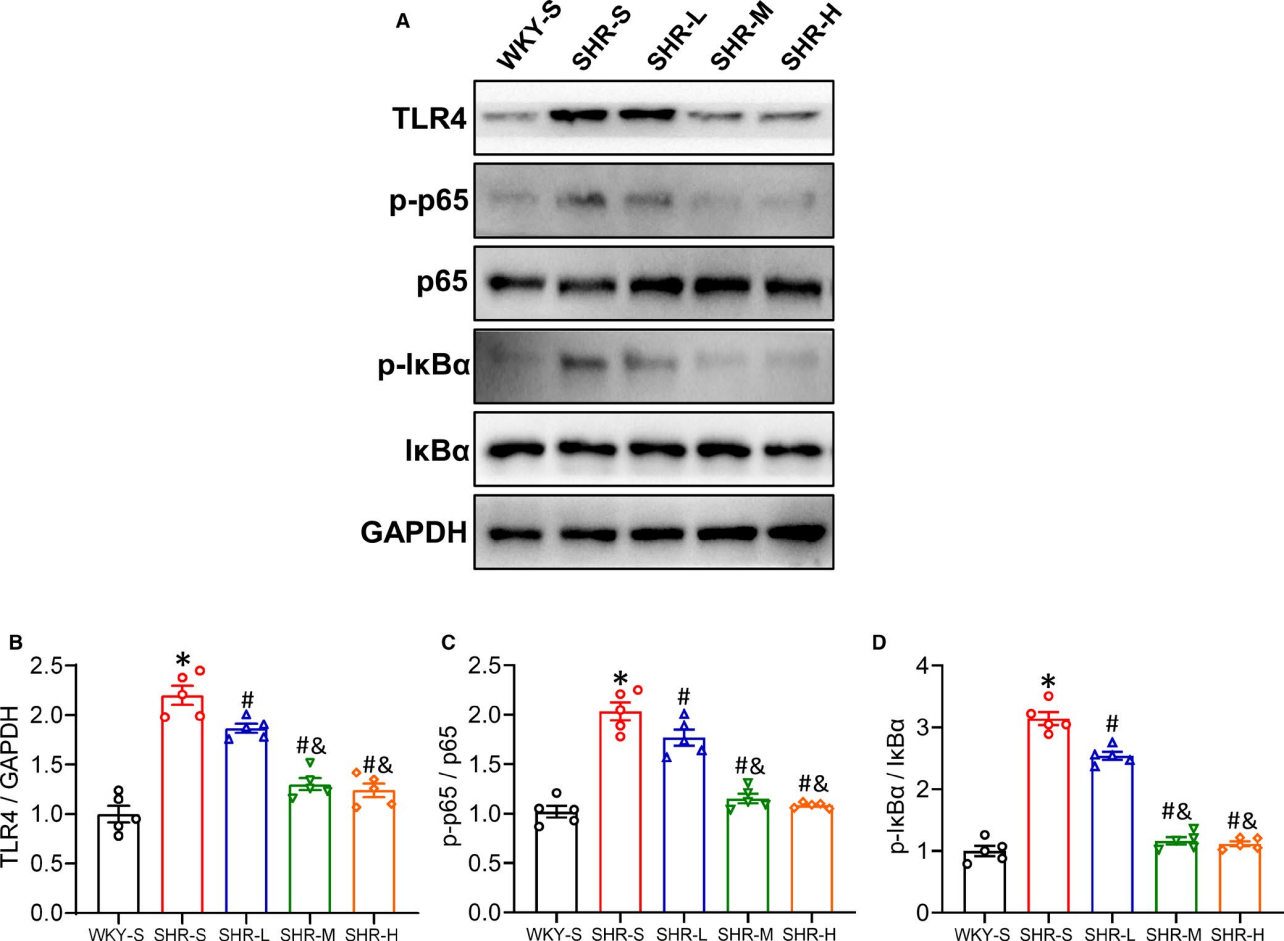
Effects of different‐intensity continuous training on TLR4/NF‐κB pathway of artery in SHR. WKY‐S, SHR‐S, SHR‐L, SHR‐M and SHR‐H were trained on the treadmill with different intensities for 14 weeks. After training, Western blot (A) was used to measure the protein expression of TLR4 (B), p‐p65 (C) and p‐IκBα (D) of the mesenteric artery. *p < 0.05 compared with WKY‐S, #*p *< 0.05 compared with SHR‐S, ^&^ *p *< 0.05 compared with SHR‐L

Studies have shown that activation of the TLR4/NF‐κB inflammatory pathway can cause vascular dysfunction in hypertension, and inhibition of this pathway can effectively improve vascular function.^4^, ^5^, ^6^, ^7^ We explored whether different training intensities affected TLR4/NF‐κB activation. We detected protein expression of TLR4, p‐p65, p65, p‐IκBα and IκBα in mesenteric arteries from different groups by Western blot (Figure 4A). Hypertension activated all the relatively detected proteins (*p *< 0.05), and activated NF‐κB pathways were inhibited by all three training paradigms (Figure 4B–D) (*p *< 0.05); the degree of inhibition increased with increasing training intensity (TLR4: SHR‐S 2.20, SHR‐L 1.87, SHR‐M 1.30, SHR‐H 1.24‐fold compared with WKY‐S; p‐p65: SHR‐S 2.04, SHR‐L 1.77, SHR‐M 1.15, SHR‐H 1.08‐fold compared with WKY‐S; p‐IκBα: SHR‐S 3.14, SHR‐L 2.54, SHR‐M 1.16, SHR‐H 1.12‐fold compared with WKY‐S).

### 3.6 Changes in NLRP3, ASC, Caspase‐1 and IL‐1β expression in the mesenteric artery

**FIGURE 5:**
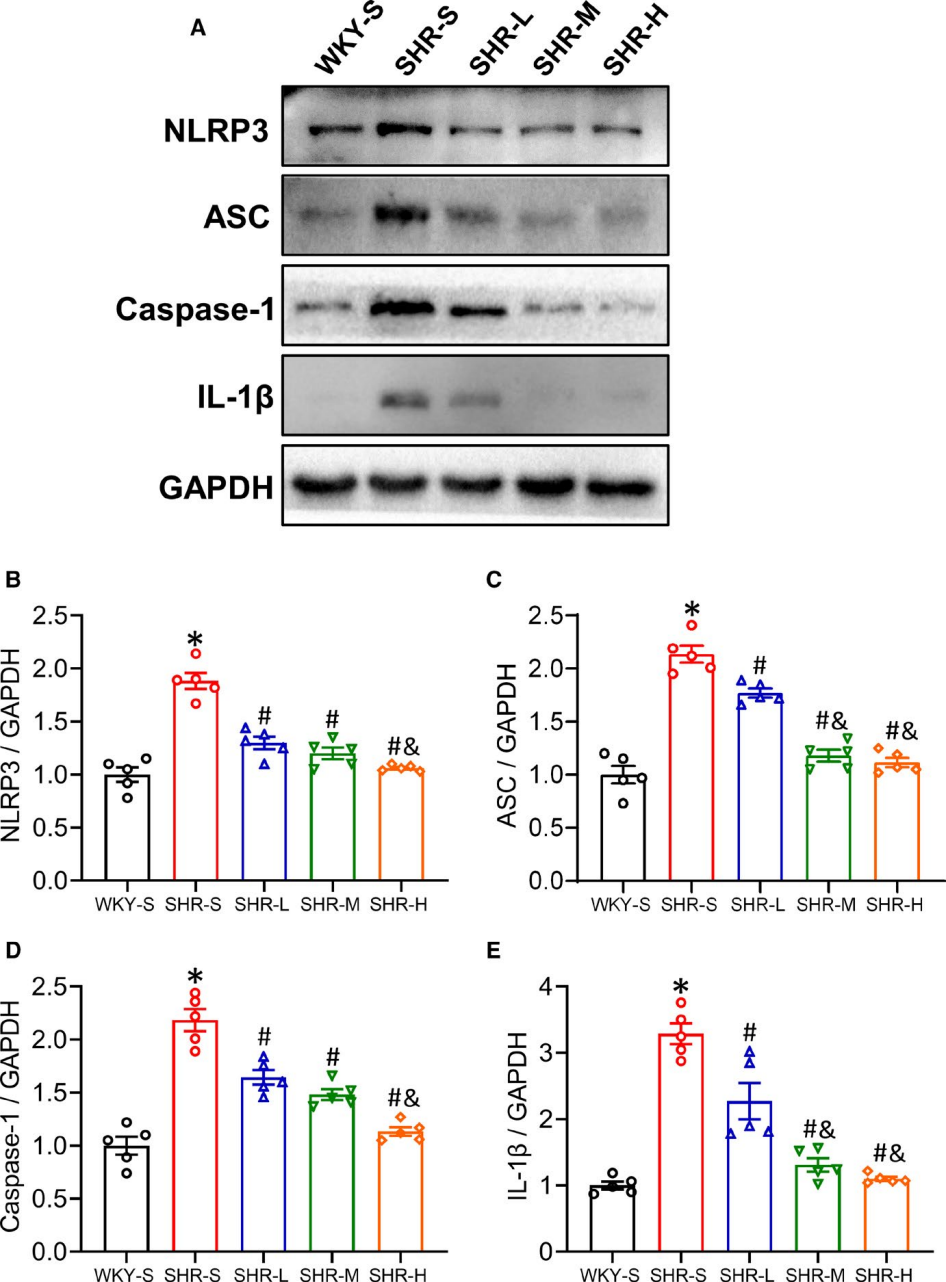
Effects of different‐intensity continuous training on NLRP3 pathway of artery in SHR. WKY‐S, SHR‐S, SHR‐L, SHR‐M and SHR‐H were trained on the treadmill with different intensities for 14 weeks. After training, Western blot (A) was used to measure the protein expression of NLRP3 (B), ASC (C), Caspase‐1 (D) and IL‐1β (E) of the mesenteric artery. *p < 0.05 compared with WKY‐S, #*p *< 0.05 compared with SHR‐S, &*p *< 0.05 compared with SHR‐L

The role of NLRP3 inflammasome in hypertensive vascular dysfunction has been confirmed; inhibition of the NLRP3 pathway improves hypertensive vascular dysfunction.^8^, ^9^, ^10^ We used Western blot to detect the expression of NLRP3, ASC, Caspase‐1 and IL‐1β in the mesenteric artery (Figure 5A). The related proteins detected in SHR‐S were activated compared with WKY‐S (*p *< 0.05), and the expression of proteins in different training intensity groups (SHR‐L, SHE‐M, SHR‐H) was lower than that in SHR‐S, indicating that training reversed the activated NLRP3 pathway, and this became more pronounced with increasing training intensity (Figure 5B–E) (*p *< 0.05) (NLRP3: SHR‐S 1.88, SHR‐L 1.30, SHR‐M 1.20, SHR‐H 1.06‐fold compared with WKY‐S; ASC: SHR‐S 2.16, SHR‐L 1.77, SHR‐M 1.18, SHR‐H 1.12‐fold compared with WKY‐S; Caspase‐1: SHR‐S 2.18, SHR‐L 1.64, SHR‐M 1.48, SHR‐H 1.13‐fold compared with WKY‐S; IL‐1β: SHR‐S 3.29, SHR‐L 2.27, SHR‐M 1.31, SHR‐H 1.10‐fold compared with WKY‐S).

### 3.7 Changes in SOD2, NOX2, NOX4 expression in mesenteric artery

**FIGURE 6:**
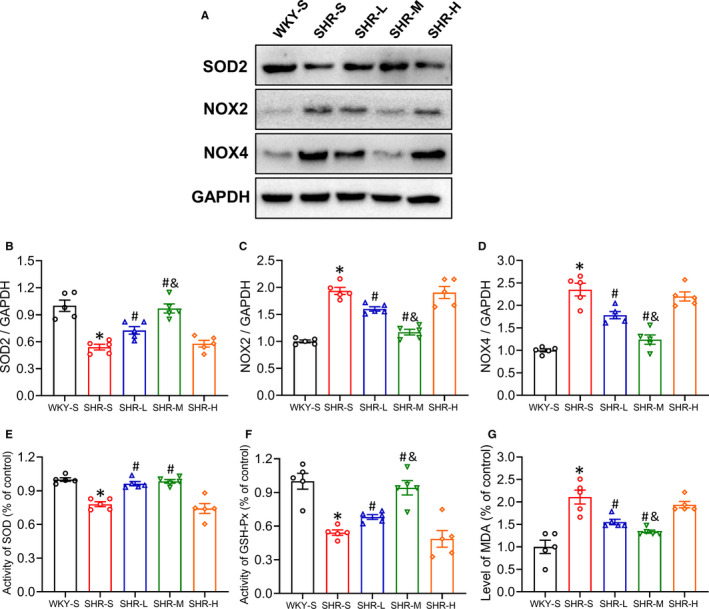
Effects of different‐intensity continuous training on SOD2, NOX2, NOX4 expression of artery and the activity of SOD, GSH‐Px and MDA level in serum in SHR. WKY‐S, SHR‐S, SHR‐L, SHR‐M and SHR‐H were trained on the treadmill with different intensities for 14 weeks. The protein expression of SOD2 (B), NOX2 (C), NOX4 (D) of the mesenteric artery and the activity of SOD (E), GSH‐Px (F) and MDA (G) level in serum were tested after training. *p < 0.05 compared with WKY‐S, #*p *< 0.05 compared with SHR‐S, &*p *< 0.05 compared with SHR‐L

Oxidative stress has been proven to damage blood vessels in the progression of hypertension. To further determine the possible mechanisms of the effect of different training intensities on vascular dysfunction in SHR, Western blot was used to detect three key enzymes involved in the regulation of oxidative stress levels: SOD2, NOX2 and NOX4. (Figure 6A). LICT and MICT significantly improved the decrease in SOD2 and the increase in NOX2 and NOX4 in mesenteric artery in SHR (*p *< 0.05), of which MICT had a better effect (*p *< 0.05), whereas there were no changes observed in SHR‐H (*p *> 0.05). The statistical values are shown in Figure 6B, C, D (SOD2: SHR‐S 0.54, SHR‐L 0.73, SHR‐M 0.97, SHR‐H 0.58‐fold compared with WKY‐S; NOX2: SHR‐S 1.94, SHR‐L 1.60, SHR‐M 1.17, SHR‐H 1.90‐fold compared with WKY‐S; NOX4: SHR‐S 2.35, SHR‐L 1.78, SHR‐M 1.24, SHR‐H 2.20‐fold compared with WKY‐S).

### 3.8 Changes in activity of SOD, GSH‐Px and MDA level in serum

To further examine the effects of chronic CT with different intensities on oxidative stress in SHR, the SOD and GSH‐Px (enzymatic defence system against free radical) activities and MDA level (indicator of lipid peroxidation) in serum were measured (Figure 6E–G). SOD and GSH‐Px activities in SHR‐S were significantly lower than that of WKY‐S (*p *< 0.05), and the SOD and GSH‐Px activities in SHR‐L and SHR‐M significantly increased compared with that in SHR‐S (*p *< 0.05). However, there was no significant change observed between SHR‐H and SHR‐S (*p *> 0.05). Additionally, the MDA level in SHR‐S was significantly higher than that in WKY‐S and significantly decreased in SHR‐L and SHR‐M compared with that in SHR‐S. However, there was no significant change observed between SHR‐H and SHR‐S regarding the MDA level (*p *> 0.05) (SOD: SHR‐S 0.78, SHR‐L 0.96, SHR‐M 0.98, SHR‐H 0.74‐fold compared with WKY‐S; GSH‐Px: SHR‐S 0.54, SHR‐L 0.68, SHR‐M 0.94, SHR‐H 0.49‐fold compared with WKY‐S; MDA: SHR‐S 2.11, SHR‐L 1.56, SHR‐M 1.34, SHR‐H 1.94‐fold compared with WKY‐S).

### 3.9 Changes in p‐AMPK, AMPK, SIRT1, SIRT3 expression in mesenteric artery

**FIGURE 7:**
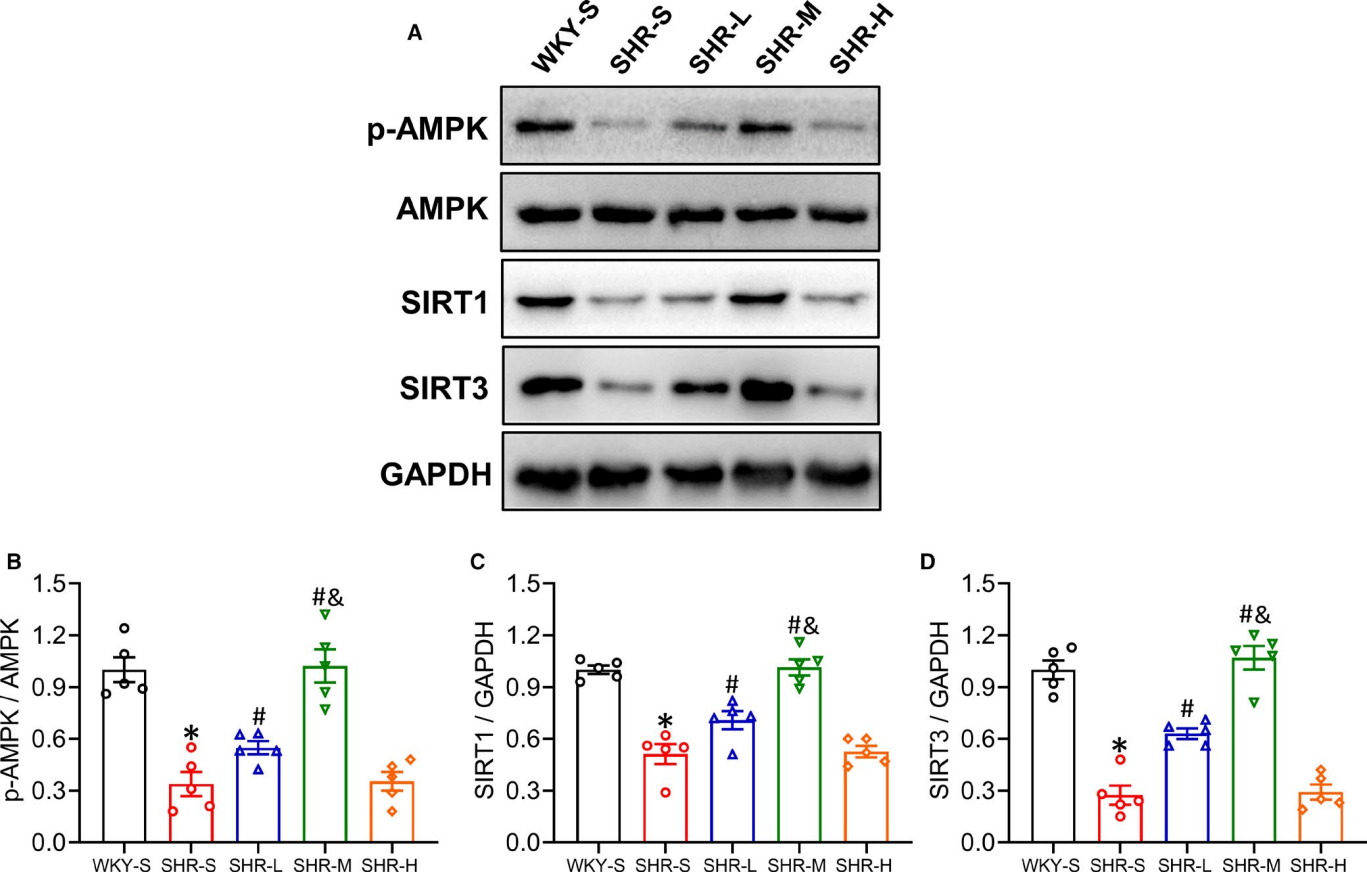
Effects of different‐intensity continuous training on p‐AMPK, SIRT1 and SIRT3 expression of artery in SHR. WKY‐S, SHR‐S, SHR‐L, SHR‐M and SHR‐H were trained on the treadmill with different intensities for 14 weeks. After training, Western blot (A) was used to measure the protein expression of p‐AMPK (B), SIRT1 (C) and SIRT3 (D) of the mesenteric artery. *p < 0.05 compared with WKY‐S, #*p *< 0.05 compared with SHR‐S, &*p *< 0.05 compared with SHR‐L

Mitochondrial dysfunction and the inhibition of energy metabolism‐related proteins, AMP‐activated protein kinase (AMPK) and Sirtuin 1/3 (SIRT1/3), play an important role in the progression of hypertension. To determine the possible mechanisms of the effect of different training intensities on SHR vascular dysfunction, we detected the protein expression of p‐AMPK, AMPK, SIRT1 and SIRT3 in different groups using Western blot (Figure 7A). LICT and MICT significantly improved the decrease in p‐AMPK, SIRT1 and SIRT3 expression in the mesenteric artery of SHR (*p *< 0.05), of which MICT had a better effect (*p *< 0.05), whereas there were no changes in SHR‐H compared with that in SHR‐S (*p *> 0.05). The statistical values are shown in Figure 7B, C and D (p‐AMPK: SHR‐S 0.34, SHR‐L 0.55, SHR‐M 1.02, SHR‐H 0.35‐fold compared with WKY‐S; SIRT1: SHR‐S 0.51, SHR‐L 0.71, SHR‐M 1.01, SHR‐H 0.53‐fold compared with WKY‐S; SIRT3: SHR‐S 0.28, SHR‐L 0.63, SHR‐M 1.07, SHR‐H 0.29‐fold compared with WKY‐S).

## 4 DISCUSSION

Our study revealed that different intensities of CT had different effects on oxidative stress and vascular inflammation in SHR. All three training paradigms could effectively reduce the activation of the TLR4/NF‐κB/NLRP3 inflammatory pathway in SHR; however, LICT and MICT could improve the blood pressure and the oxidative stress of SHR, whereas HICT did not have a positive effect on blood pressure and oxidative stress.

There is evidence that different training intensities have different effects on the vascular function of hypertensive animal models.^32^, ^33^, ^34^, ^35^ Appropriate training intensity is increasingly becoming the key to training therapy. Inflammatory response and oxidative stress have been extensively studied in SHR. This research aimed to explore the effects of different intensities of training (35% VO_2_max, 50% VO_2_max, 65% VO_2_max) on inflammation and oxidative stress in blood vessels of SHR.

**FIGURE 8:**
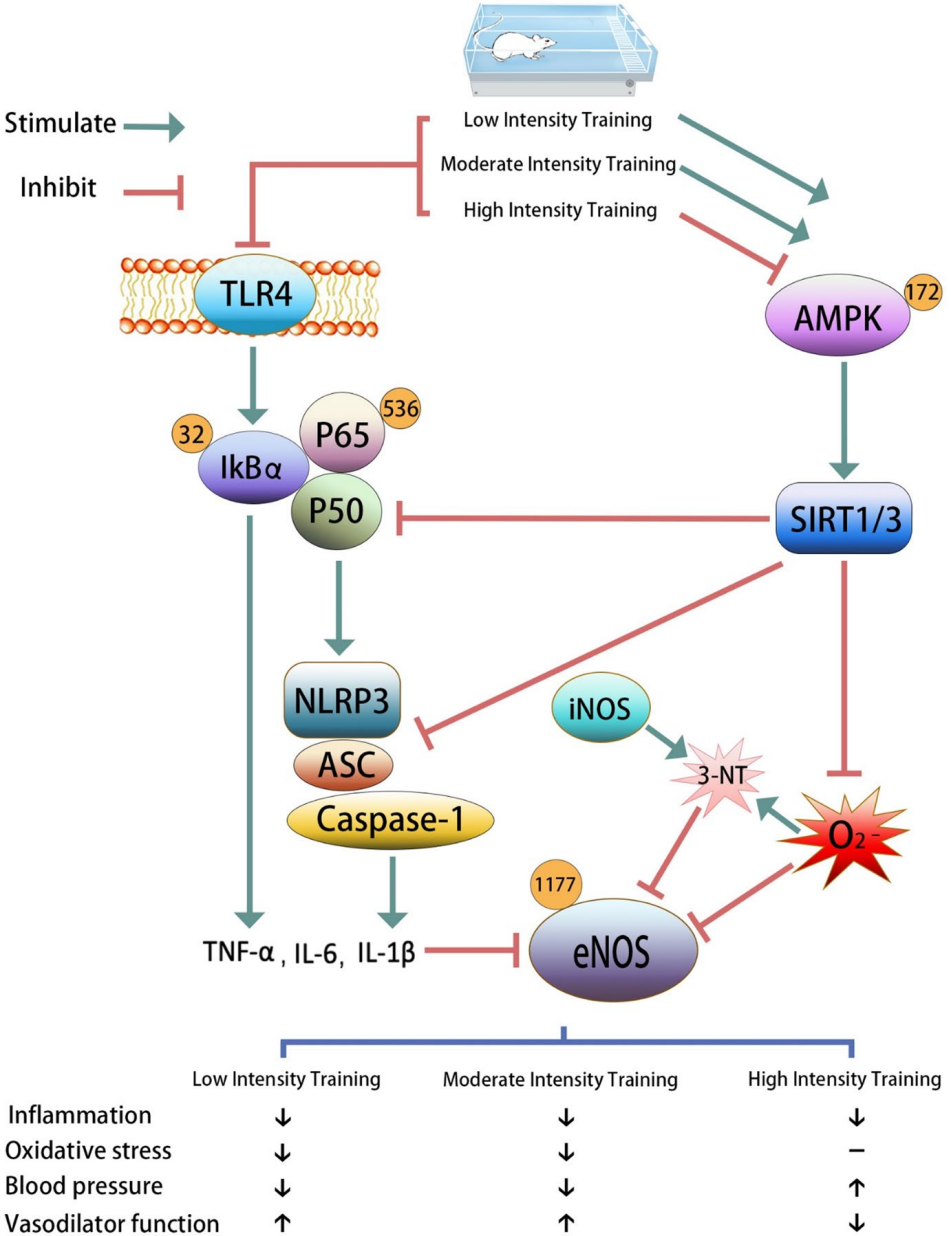
Graphic abstract

Seven key findings were observed in this research (Figure 8). (1) HICT led to an increase in the blood pressure of SHR (mainly DBP), whereas LICT and MICT reduced the blood pressure of SHR; overall, MICT reduced the blood pressure more significantly. (2) HICT did not improve the intima‐media thickness of SHR aortic vessels. LICT and MICT improved the morphological changes of SHR aorta; MICT showed more significant results. (3) All three training paradigms could significantly improve the expression of eNOS and iNOS changes in SHR mesenteric artery; MICT showed the best effect. However, the p‐eNOS‐Ser1177 expression decreased in SHR‐H compared with that in SHR‐S. (4) HICT impaired the ACh‐induced relaxation function of SHR aortic vessels. LICT and MICT improved the ACh‐induced relaxation function of SHR aorta; MICT showed more significant results. (5) All three training paradigms could significantly improve the TLR4/NF‐κB/NLRP3 inflammatory pathway change in the mesenteric arteries of SHR; HICT showed the best effect. (6) HICT had no effect on antioxidant and oxidative stress indicators in SHR, whereas LICT and MICT significantly improved these indicators; MICT showed more significant improvement. (7) LICT and MICT significantly improved the expression changes of p‐AMPK, SIRT1 and SIRT3 in the mesenteric arteries of SHR; MICT showed better effects. However, there were no significant changes in SHR‐H compared with SHR‐S.

Some explanations for the reduction of blood pressure through training include neurohumoral adaptation, vascular structural adaptation, reduction of catecholamines and total peripheral resistance, increased insulin sensitivity and changes in vasodilators and vasoconstrictors.^36^, ^37^ Nevertheless, it is not yet possible to clearly elucidate the mechanism of blood pressure reduction after endurance training. This study found that different intensities of CT induce different adaptability changes to the vascular structure and function. Compared with WKY‐S, endothelial functions in the thoracic aortas of SHR‐S were impaired, and the intima‐media was thickened. In LICT and MICT, endothelium‐dependent diastolic function improved and the thickness of intima‐media layer decreased, but there was no significant difference between the SHR‐H and SHR‐S. Our results suggested that LICT and MICT had significant effects on blood pressure, including the SBP and DBP. Moderate training had a more significant effect in reducing blood pressure, whereas HICT further increased the DBP in SHR. DBP is closely related to peripheral vascular resistance, suggesting that HICT may cause damage to peripheral resistance blood vessels. In this study, we examined proteins associated with inflammation and oxidative stress in mesenteric artery rather than aorta, considering that the vascular function of small blood vessels or resistance vessels can better reflect the real peripheral resistance blood vessels in various organs of the body. However, considering that vascular morphometry analysis and vascular reactivity experiment about the secondary branches of mesenteric artery are difficult, we only conducted these tests in the aorta.

Early studies reported that the mechanism by which training improves blood pressure is related to the eNOS‐NO pathway.^38^ NO in vascular endothelial cells plays an important role in angiogenesis, regulating vascular tension, inhibiting smooth muscle cell proliferation and migration, and inhibiting platelet aggregation and adhesion.^38^ Decreased level of NO produced by eNOS in vascular endothelial cells is a hallmark of endothelial dysfunction, characterized by impaired endothelium‐dependent relaxation, which is an early marker for hypertension. The results of this study found that eNOS expression was up‐regulated in training groups with different intensities, but the blood pressure and vascular morphology in the SHR‐H showed no positive changes. We speculated that HICT caused eNOS uncoupling and caused inhibition of the eNOS function.^39^ In the past few years, iNOS has been reported to be mainly induced by cytokines, which is closely related to the vascular dysfunction of hypertension.^40^ We observed that the vascular iNOS expression in SHR‐S rats was significantly higher than that in WKY‐S rats and chronic CT could reduce the expression of iNOS in vascular tissues.

Acute high‐intensity muscle training promotes the production of ROS, which is related to oxidative damage and the activation of various biochemical signalling pathways.^41^ HICT causes an increase in the level of vascular oxidative stress, leading to an increase in the ROS‐related uncoupling of eNOS, and the balance between NO and ROS is lost, which in turn causes a vicious cycle of reduced NO bioavailability and increased ROS.^42^ In human studies, it has been confirmed that high‐intensity training increases oxidative stress.^41^, ^42^, ^43^, ^44^ The superoxide radical O_2_^−^ produced through high‐intensity training reacts with NO and converts into peroxynitrite (ONOO^−^), and the production of ONOO^−^ reduces the bioavailability of NO,^44^ which may be involved in the progression of oxidative stress in SHR; our data regarding the detection of 3‐NT in the mesenteric artery further confirmed this conclusion. There are two free radical (FR) defence systems in the human body: an enzymatic defence system involving superoxide dismutase, glutathione peroxidase (GSH‐Px), catalase (CAT) and glutathione reductase (GR), and a non‐enzymatic defence system involving vitamin C, vitamin E and glutathione (GSH). Typically, the body keeps a dynamic balance between the generation and the removal of FR. When the level of lipid peroxidation exceeds the body's antioxidant capacity, such as malondialdehyde (MDA), it induces oxidative stress and directly causes biofilm injury, the degeneration of intracellular proteins, leading to cell death, apoptosis, tissue damage and disease. This study did not directly measure the content of ROS in blood vessels but observed the activity of SOD, GSH‐Px and MDA level, and the protein expression of SOD2, NOX2 and NOX4, which indirectly proved that CT of different intensities had an effect on the oxidative stress level of blood vessels in SHR. Our results revealed that high‐intensity training had no effect on the activity of SOD and GSH‐Px, MDA level in serum, and SOD2, NOX2 and NOX4 expression in the mesenteric arteries of SHR, whereas low‐ and medium‐intensity training could significantly improve oxidative stress. Previous studies have investigated the relationship between CT and oxidative stress level in vivo by measuring the SOD and GSH‐Px activities and the MDA level.^45^, ^46^, ^47^

Although many studies have shown that CT can reduce oxidative stress, there is a paradox in the relationship between training and the production of ROS. Training can also induce oxidative stress under certain conditions, such as acute high‐intensity training and ultra‐marathon training. Additionally, appropriate training may cause the body to be exposed to mild oxidative stress repeatedly, which may initiate an adaptive process. Promoting antioxidant enzymes to reduce O_2_^−^ to subside oxidative stress may help increase the supply of physiological NO during training. Human studies have shown that high‐intensity interval training (HIIT) is more effective than MICT in improving arterial vascular function and can positively affect cardiopulmonary health, traditional cardiovascular disease risk factors, oxidative stress, inflammation and insulin sensitivity.^48^ HIIT was found to induce a significant enhancement in the antioxidant status.^49^ In our study, HICT can effectively improve the inflammatory response in SHR but does not improve oxidative stress. We believe that the inconsistency between the results observed by HIIT and HICT may arise due to the difference in oxidative stress. However, the current research is not sufficient to draw conclusions as to why HIIT and HICT have opposite effects on hypertension.

Activation of the NLRP3 inflammasome in endothelial cells under pathophysiological conditions may aggravate endothelial dysfunction, leading to various diseases.^50^, ^51^, ^52^ The activation of NF‐κB signalling pathways is well confirmed in cardiovascular system dysfunction in hypertensive models.^53^ The research results suggest that all three training intensities have significant inhibitory effects on NF‐κB and NLRP3 signalling pathways in SHR resistance vascular tissue. Chronic low‐grade inflammation of the resistance vessels is a characteristic of hypertension. The levels of pro‐inflammatory factors such as TNF‐α, IL‐1β and IL‐6 increase with the severity of hypertension,^54^ and affect its prognosis. The inhibitory effect of training on inflammatory factors has been reported.^55^ This study reported the role of training in regulating NF‐κB and NLRP3 pathways in SHR for the first time. Although high‐intensity training subsided the vascular inflammation in SHR, the vascular morphology and blood pressure in SHR‐H rats did not improve. Our results suggested that the difference in the effects of different intensities of CT on the blood vessels of hypertensive rats may not be the result of inflammation. A previous study indicated that the increase of inflammatory factors after strenuous training was a systemic result owing to the depletion of muscle glycogen and inflammatory markers reduced after 24 h of training.^56^ However, other studies have shown that the level of plasma inflammatory cells increased after training, but the gene expression of IL‐1β, IL‐6 and TNF‐α did not increase.^57^

AMP‐activated protein kinase is a regulator of systemic energy metabolism and a candidate signal regulator of PGC‐1α and SIRT1/3 in mitochondrial biosynthesis and homeostasis.^58^ Sirtuins act as cell sensors to detect energy supply and regulate metabolic processes. Mammalian sirtuin 1 and sirtuin 3 are the two cores that control metabolic processes and are located in the nucleus and mitochondria, respectively.^59^ Changes in the function of SIRT1 and SIRT3 significantly affect the vascular function associated with hypertension.^60^, ^61^ AMPK‐SIRT1/3 inhibition can lead to mitochondrial damage followed by mitochondrial oxidative stress caused by SOD2 inactivation. At the same time, the release of mitochondrial DNA can directly activate the NLRP3 inflammasome, leading to the activation of caspase‐1, the release of IL‐1β and the formation of MCP1.^62^ It can also activate the redox‐dependent pro‐inflammatory transcription factor NF‐kB. This study used Western blots to directly observe the TLR4/NF‐κB/NLRP3, AMPK‐SIRT1/3 pathway and oxidative stress‐related proteins. Our results showed that low‐intensity training and medium‐intensity training could significantly improve the expression of AMPK‐SIRT1/SIRT3 in the mesenteric arteries of SHR. Chronic regular low‐intensity and medium‐intensity training delayed the pathophysiological progression of hypertension, reduced blood pressure and improved the vascular function of hypertensive rats. More importantly, this study provided evidence that the antihypertensive and vascular protective effects of chronic training were achieved by down‐regulating NF‐κB and NLRP3, improving cell redox homeostasis and increasing NO production. Although no direct causality can be established in this study, we may attribute the beneficial effects of training with lower than moderate intensities on hypertension to the changes in the interaction between sympathetic nerve activity, inflammation, and oxidative stress. Future research may aim to provide more direct evidence to support the causal relationship between various parameters.

Interestingly, our data suggested that the HICT and the MICT have opposite results regarding oxidative stress and AMPK‐SIRT1/3. There are enough studies to prove that training increases the oxygen consumption of mitochondria, leading to an increase in the production of ROS,^63^ and proper exercise can effectively reduce inflammation and oxidative stress in hypertension.^64^ Excessive exercise promotes oxidative stress.^63^ Reactive nitrogen and oxygen compounds’ (RONS) levels can influence the phosphorylation of Ser485‐AMPKα1/Ser491‐AMPKα2, thereby inhibiting the phosphorylation of Thr172‐AMPKα,^65^ and excessive RONS production reduces Thr172‐AMPKα phosphorylation during hypoxia.^66^ Another important mechanism may be that the accumulation of lactic acid produced by long‐term high‐intensity exercise is accompanied by a decrease in pH, and the relative abundance of AMPK decreases in a long‐term low‐pH environment.^67^, ^68^ Thr172‐AMPKα phosphorylation is reduced, consequently reducing mitochondrial biogenesis, which is not conducive to the increase of functional protein PGC1α. We speculated this change might be one of the important reasons why AMPK function was inhibited in SHR rats with high‐intensity training in our study; we plan to conduct further research on this topic.

Among the three intensities we set: LICT (35% VO_2_max), MICT (50% VO_2_max) and HICT (65% VO_2_max), we believe that there existed a certain beneficial training intensity between 50%–65% VO_2_max in aerobic training, and training intensity higher than this value is not suitable to treat hypertension.

In conclusion, this study showed that CT with different intensities had different effects on oxidative stress and vascular inflammation in SHR. All three training paradigms could effectively reduce the activation of the TLR4/NF‐κB/NLRP3 inflammatory pathway in SHR, but only low‐intensity and medium‐intensity training could improve the blood pressure and the oxidative stress in SHR, whereas high‐intensity training did not show any positive effects. Therefore, we suggest choosing medium‐ or low‐intensity (≤50% VO_2_max) continuous exercise training as a therapeutic strategy for hypertension patients. Future research on the relationship between training intensity and hypertension should be conducted to further explore the critical point between medium‐intensity and high‐intensity training and to explain the differences in the changes to oxidative stress caused by different intensities of exercise.

## CONFLICT OF INTEREST

The authors declare no conflict of interest.

## AUTHOR CONTRIBUTIONS

**guochun liu:** Conceptualization (equal); Data curation (equal); Formal analysis (equal); Funding acquisition (lead); Investigation (equal); Methodology (equal); Project administration (equal). **Minghao Luo:** Conceptualization (equal); Data curation (equal); Formal analysis (equal); Investigation (equal); Methodology (equal); Project administration (equal). **Chun‐mei Cao:** Formal analysis (equal); Methodology (equal). **Josef Niebauer:** Investigation (equal). **Jiang‐hong Yan:** Formal analysis (equal); Investigation (equal). **Xin‐dong Ma:** Funding acquisition (equal); Investigation (equal). **qing chang:** Funding acquisition (supporting); Investigation (equal). **Ting Zhang:** Data curation (equal); Formal analysis (equal). **Xiaoxiao huang:** Investigation (equal).

## Data Availability

All data utilized in this study are included in this article, and all data supporting the findings of this study are available on reasonable request from the corresponding author.

## References

[1] Steven S, Frenis K, Oelze M, et al. Vascular inflammation and oxidative stress: major triggers for cardiovascular disease. Oxid Med Cell Longev. 2019;2019:7092151‐7092226. 31341533 10.1155/2019/7092151 PMC6612399

[2] Tanito M, Nakamura H, Kwon YW, et al. Enhanced oxidative stress and impaired thioredoxin expression in spontaneously hypertensive rats. Antioxid Redox Signal. 2004;6:89‐97. 14713339 10.1089/152308604771978381

[3] Siti HN, Kamisah Y, Kamsiah J. The role of oxidative stress, antioxidants and vascular inflammation in cardiovascular disease (a review). Vascul Pharmacol. 2015;71:40‐56. 25869516 10.1016/j.vph.2015.03.005

[4] Hernanz R, Martínez‐Revelles S, Palacios R, et al. Toll‐like receptor 4 contributes to vascular remodelling and endothelial dysfunction in angiotensin II‐induced hypertension. Br J Pharmacol. 2015;172:3159‐3176. 25712370 10.1111/bph.13117 PMC4459031

[5] Hayden MS, Ghosh S. Shared principles in NF‐kappaB signaling. Cell. 2008;132(3):344‐362. 18267068 10.1016/j.cell.2008.01.020

[6] Kumar A, Takada Y, Boriek AM, et al. Nuclear factor‐kappaB: its role in health and disease. J Mol Med (Berl). 2004;82(7):434‐448. 15175863 10.1007/s00109-004-0555-y

[7] Queisser N, Schupp N. Aldosterone, oxidative stress, and NF‐kappaB activation in hypertension‐related cardiovascular and renal diseases. Free Radic Biol Med. 2012;53(2):314‐327. 22609249 10.1016/j.freeradbiomed.2012.05.011

[8] Jaén RI, Val‐Blasco A, Prieto P, et al. Innate immune receptors, key actors in cardiovascular diseases. JACC Basic Transl Sci. 2020;5(7):735‐749. 32760860 10.1016/j.jacbts.2020.03.015 PMC7393405

[9] Hoseini Z, Sepahvand F, Rashidi B, et al. NLRP3 inflammasome: Its regulation and involvement in atherosclerosis. J Cell Physiol. 2018;233(3):2116‐2132. 28345767 10.1002/jcp.25930

[10] Fusco R, Siracusa R, Genovese T, et al. Focus on the role of NLRP3 inflammasome in diseases. Int J Mol Sci. 2020;21(12):4223. 10.3390/ijms21124223 PMC7352196 32545788

[11] Cai H, Harrison DG. Endothelial dysfunction in cardiovascular diseases: the role of oxidant stress. Circ Res. 2000;87(10):840‐844. 11073878 10.1161/01.res.87.10.840

[12] Brown DI, Griendling KK. Regulation of signal transduction by reactive oxygen species in the cardiovascular system. Circ Res. 2015;116(3):531‐549. 25634975 10.1161/CIRCRESAHA.116.303584 PMC4392388

[13] Montezano AC, Tsiropoulou S, Dulak‐Lis M, et al. Redox signaling, Nox5 and vascular remodeling in hypertension. Curr Opin Nephrol Hypertens. 2015;24(5):425‐433. 26197203 10.1097/MNH.0000000000000153 PMC4727501

[14] Laurindo FR, Araujo TL, Abrahao TB. Nox NADPH oxidases and the endoplasmic reticulum. Antioxid Redox Signal. 2014;20(17):2755‐2775. 24386930 10.1089/ars.2013.5605 PMC4026305

[15] Fukai T, Ushio‐Fukai M. Superoxide dismutases: role in redox signaling, vascular function, and diseases. Antioxid Redox Signal. 2011;15:1583‐1606. 21473702 10.1089/ars.2011.3999 PMC3151424

[16] Joyner MJ, Green DJ. Exercise protects the cardiovascular system: effects beyond traditional risk factors. J Physiol. 2009;587(Pt 23):5551‐5558. 19736305 10.1113/jphysiol.2009.179432 PMC2805367

[17] Cornelissen VA, Smart NA. Exercise training for blood pressure: a systematic review and meta‐analysis. J Am Heart Assoc. 2013;2(1):e004473. 23525435 10.1161/JAHA.112.004473 PMC3603230

[18] Zhang L, Wang XJ, Zhang H, et al. Exercise‐induced peptide EIP‐22 protect myocardial from ischaemia/reperfusion injury via activating JAK2/STAT3 signalling pathway. J Cell Mol Med. 2021;25(7):3560‐3572. 33710777 10.1111/jcmm.16441 PMC8034444

[19] Green DJ, Smith KJ. Effects of exercise on vascular function, structure, and health in humans. Cold Spring Harb Perspect Med. 2018;8(4):A029819. 28432115 10.1101/cshperspect.a029819 PMC5880156

[20] Thompson PD, Buchner D, Pina IL, et al. Exercise and physical activity in the prevention and treatment of atherosclerotic cardiovascular disease: a statement from the council on clinical cardiology (subcommittee on exercise, rehabilitation, and prevention) and the council on nutrition, physical activity, and metabolism (subcommittee on physical activity). Circulation. 2003;107(24):3109‐3116. 12821592 10.1161/01.CIR.0000075572.40158.77

[21] Wang R, Tian H, Guo D, et al. Impacts of exercise intervention on various diseases in rats. J Sport Health Sci. 2020;9:211‐227. 32444146 10.1016/j.jshs.2019.09.008 PMC7242221

[22] Naderi‐Boldaji V, Joukar S, Noorafshan A, et al. The effect of blood flow restriction along with low‐intensity exercise on cardiac structure and function in aging rat: role of angiogenesis. Life Sci. 2018;209:202‐209. 30096385 10.1016/j.lfs.2018.08.015

[23] Eaton M, Granata C, Barry J, et al. Impact of a single bout of high‐intensity interval exercise and short‐term interval training on interleukin‐6, FNDC5, and METRNL mRNA expression in human skeletal muscle. J Sport Health Sci. 2018;7:191‐196. 30356443 10.1016/j.jshs.2017.01.003 PMC6180539

[24] Bertagnolli M, Schenkel PC, Campos C, et al. Exercise training reduces sympathetic modulation on cardiovascular system and cardiac oxidative stress in spontaneously hypertensive rats. Am J Hypertension. 2008;21:1188‐1193. 10.1038/ajh.2008.270 18787517

[25] Agarwal D, Haque M, Sriramula S, et al. Role of proinflammatory cytokines and redox homeostasis in exercise‐induced delayed progression of hypertension in spontaneously hypertensive rats. Hypertension. 2009;54(6):1393‐1400. 19841289 10.1161/HYPERTENSIONAHA.109.135459 PMC2780026

[26] Kilic‐Erkek O, Kilic‐Toprak E, Caliskan S, et al. Detraining reverses exercise‐induced improvement in blood pressure associated with decrements of oxidative stress in various tissues in spontaneously hypertensive rats. Mol Cell Biochem. 2016;412:209‐219. 26708216 10.1007/s11010-015-2627-4

[27] Di Francescomarino S, Sciartilli A, Di Valerio V, et al. The effect of physical exercise on endothelial function. Sports Med. 2009;39(10):797‐812. 19757859 10.2165/11317750-000000000-00000

[28] Ulrik Wisloff JH, Kemi OJ, Ellingsen O. Intensity‐controlled treadmill running in rats: Vo2max and cardiac hypertrophy. Am J Physiol. 2001;49(3):H1301. 10.1152/ajpheart.2001.280.3.H1301 11179077

[29] Poole DC, Copp SW, Colburn TD, et al. Guidelines for animal exercise and training protocols for cardiovascular studies. Am J Physiol Heart Circ Physiol. 2020;318(5):H1100‐H1138. 32196357 10.1152/ajpheart.00697.2019 PMC7254566

[30] Luo M, Luo S, Cheng Z, et al. Tubeimoside I improves survival of mice in sepsis by inhibiting inducible nitric oxide synthase expression. Biomed Pharmacother. 2020;126:110083. 32272432 10.1016/j.biopha.2020.110083

[31] Luo M, Meng J, Yan J, et al. Role of the nucleotide‐binding domain‐like receptor protein 3 inflammasome in the endothelial dysfunction of early sepsis. Inflammation. 2020;43:1561‐1571. 32239396 10.1007/s10753-020-01232-x

[32] Chen Y, Zhang H, Zhang Y, et al. Exercise intensity‐dependent reverse and adverse remodeling of voltage‐gated Ca(2+) channels in mesenteric arteries from spontaneously hypertensive rats. Hypertens Res. 2015;38(10):656‐665. 25902901 10.1038/hr.2015.56

[33] Battault S, Singh F, Gayrard S, et al. Endothelial function does not improve with high‐intensity continuous exercise training in SHR: implications of eNOS uncoupling. Hypertens Res. 2016;39(2):70‐78. 26537830 10.1038/hr.2015.114

[34] Ye F, Wu Y, Chen Y, et al. Impact of moderate‐ and high‐intensity exercise on the endothelial ultrastructure and function in mesenteric arteries from hypertensive rats. Life Sci. 2019;222:36‐45. 30825543 10.1016/j.lfs.2019.01.058 PMC6497179

[35] Krzesiak A, Cognard C, Sebille S, et al. High‐intensity intermittent training is as effective as moderate continuous training, and not deleterious, in cardiomyocyte remodeling of hypertensive rats. J Appl Physiol (1985). 2019;126(4):903‐915. 30702976 10.1152/japplphysiol.00131.2018

[36] Valenzuela PL, Carrera‐Bastos P, Gálvez BG, et al. Lifestyle interventions for the prevention and treatment of hypertension. Nat Rev Cardiol. 2021;18(4):251‐275. 33037326 10.1038/s41569-020-00437-9

[37] Pescatello LS, Buchner DM, Jakicic JM, et al. Physical activity to prevent and treat hypertension: a systematic review. Med Sci Sports Exerc. 2019;51:1314‐1323. 31095088 10.1249/MSS.0000000000001943

[38] Radomski MW, Palmer RM, Moncada S. Endogenous nitric oxide inhibits human platelet adhesion to vascular endothelium. Lancet. 1987;300(8567):1057‐1058. 10.1016/s0140-6736(87)91481-4 2889967

[39] Farah C, Kleindienst A, Bolea G, et al. Exercise‐induced cardioprotection: a role for eNOS uncoupling and NO metabolites. Basic Res Cardiol. 2013;108:389. 24105420 10.1007/s00395-013-0389-2

[40] Hong HJ, Loh SH, Yen MH. Suppression of the development of hypertension by the inhibitor of inducible nitric oxide synthase. Br J Pharmacol. 2000;131(3):631‐637. 11015317 10.1038/sj.bjp.0703603 PMC1572360

[41] Powers SK, Deminice R, Ozdemir M, et al. Exercise‐induced oxidative stress: friend or foe? J Sport Health Sci. 2020;9(5):415‐425. 32380253 10.1016/j.jshs.2020.04.001 PMC7498668

[42] Higashi Y. Exercise is a double‐edged sword for endothelial function. Hypertens Res. 2016;39(2):61‐63. 26559608 10.1038/hr.2015.127

[43] Vollaard NB, Shearman JP, Cooper CE. Exercise‐induced oxidative stress: myths, realities and physiological relevance. Sports Med. 2005;35(12):1045‐1062. 16336008 10.2165/00007256-200535120-00004

[44] Gholami F, Rahmani L, Amirnezhad F, et al. High doses of sodium nitrate prior to exhaustive exercise increases plasma peroxynitrite levels in well‐trained subjects: randomized, double‐blinded, crossover study. Appl Physiol Nutr Metab. 2019;44(12):1305‐1310. 31051087 10.1139/apnm-2018-0816

[45] Liu Z, Nie R, Liu Y, et al. Effects of total soy saponins on free radicals in the quadriceps femoris, serum testosterone, LDH, and BUN of exhausted rats. J Sport Health Sci. 2017;6(3):359‐364. 30356590 10.1016/j.jshs.2016.01.016 PMC6189000

[46] Wang X, Qu Y, Zhang Y, et al. Antifatigue potential activity of sarcodon imbricatus in acute excise‐treated and chronic fatigue syndrome in mice via regulation of Nrf2‐mediated oxidative stress. Oxid Med Cell Longev. 2018;2018:9140896‐9140913. 30050662 10.1155/2018/9140896 PMC6046126

[47] Husain K. Interaction of physical training and chronic nitroglycerin treatment on blood pressure, nitric oxide, and oxidants/antioxidants in the rat heart. Pharmacol Res. 2003;48(3):253‐261. 12860443 10.1016/s1043-6618(03)00150-6

[48] Ramos JS, Dalleck LC, Tjonna AE, et al. The impact of high‐intensity interval training versus moderate‐intensity continuous training on vascular function: a systematic review and meta‐analysis. Sports Med. 2015;45(5):679‐692. 25771785 10.1007/s40279-015-0321-z

[49] Mitranun W, Deerochanawong C, Tanaka H, et al. Continuous vs interval training on glycemic control and macro‐ and microvascular reactivity in type 2 diabetic patients. Scand J Med Sci Sports. 2014;24(2):e69‐e76. 24102912 10.1111/sms.12112

[50] Bai B, Yang Y, Wang Q, et al. NLRP3 inflammasome in endothelial dysfunction. Cell Death Dis. 2020;11:776. 32948742 10.1038/s41419-020-02985-x PMC7501262

[51] Bruder‐Nascimento T, Ferreira NS, Zanotto CZ, et al. NLRP3 inflammasome mediates aldosterone‐induced vascular damage. Circulation. 2016;134:1866‐1880. 27803035 10.1161/CIRCULATIONAHA.116.024369

[52] Liu D, Zeng X, Li X, et al. Role of NLRP3 inflammasome in the pathogenesis of cardiovascular diseases. Basic Res Cardiol. 2018;113(1):5. 29224086 10.1007/s00395-017-0663-9

[53] Sun SC. The non‐canonical NF‐kappaB pathway in immunity and inflammation. Nat Rev Immunol. 2017;17(9):545‐558. 28580957 10.1038/nri.2017.52 PMC5753586

[54] Peeters AC, Netea MG, Janssen MC, et al. Pro‐inflammatory cytokines in patients with essential hypertension. Eur J Clin Invest. 2001;31(1):31‐36. 11168436 10.1046/j.1365-2362.2001.00743.x

[55] Petersen AM, Pedersen BK. The anti‐inflammatory effect of exercise. J Appl Physiol (1985). 2005;98(4):1154‐1162. 15772055 10.1152/japplphysiol.00164.2004

[56] Scherr J, Braun S, Schuster T, et al. 72‐h kinetics of high‐sensitive troponin T and inflammatory markers after marathon. Med Sci Sports Exerc. 2011;43:1819‐1827. 21448080 10.1249/MSS.0b013e31821b12eb

[57] Moldoveanu AI, Shephard RJ, Shek PN. Exercise elevates plasma levels but not gene expression of IL‐1β, IL‐6, and TNF‐α in blood mononuclear cells. J Appl Physiol. 2000;89(4):1499‐1504. 11007588 10.1152/jappl.2000.89.4.1499

[58] Duan WJ, Li YF, Liu FL, et al. A SIRT3/AMPK/autophagy network orchestrates the protective effects of trans‐resveratrol in stressed peritoneal macrophages and RAW 264.7 macrophages. Free Radic Biol Med. 2016;95:230‐242. 27021965 10.1016/j.freeradbiomed.2016.03.022

[59] Nogueiras R, Habegger KM, Chaudhary N, et al. Sirtuin 1 and sirtuin 3: physiological modulators of metabolism. Physiol Rev. 2012;92:1479‐1514. 22811431 10.1152/physrev.00022.2011 PMC3746174

[60] Wang F, Chen HZ. Histone deacetylase SIRT1, smooth muscle cell function, and vascular diseases. Front Pharmacol. 2020;11:537519. 33117155 10.3389/fphar.2020.537519 PMC7573826

[61] Dikalova AE, Pandey A, Xiao L, et al. Mitochondrial deacetylase Sirt3 reduces vascular dysfunction and hypertension while Sirt3 depletion in essential hypertension is linked to vascular inflammation and oxidative stress. Circ Res. 2020;126:439‐452. 31852393 10.1161/CIRCRESAHA.119.315767 PMC7035170

[62] Traba J, Geiger SS, Kwarteng‐Siaw M, et al. Prolonged fasting suppresses mitochondrial NLRP3 inflammasome assembly and activation via SIRT3‐mediated activation of superoxide dismutase 2. J Biol Chem. 2017;292:12153‐12164. 28584055 10.1074/jbc.M117.791715 PMC5519366

[63] Powers SK, Radak Z, Ji LL. Exercise‐induced oxidative stress: past, present and future. J Physiol. 2016;594(18):5081‐5092. 26893258 10.1113/JP270646 PMC5023699

[64] Barhoumi T, Briet M, Kasal DA, et al. Erythropoietin‐induced hypertension and vascular injury in mice overexpressing human endothelin‐1: exercise attenuated hypertension, oxidative stress, inflammation and immune response. J Hypertens. 2014;32:784‐794. 24463938 10.1097/HJH.0000000000000101

[65] Morales‐Alamo D, Ponce‐González JG, Guadalupe‐Grau A, et al. Increased oxidative stress and anaerobic energy release, but blunted Thr172‐AMPKα phosphorylation, in response to sprint exercise in severe acute hypoxia in humans. J Appl Physiol. 2012;113(06):917‐928. 22858621 10.1152/japplphysiol.00415.2012

[66] Morales‐Alamo D, Calbet JAL. AMPK signaling in skeletal muscle during exercise: role of reactive oxygen and nitrogen species. Free Radic Biol Med. 2016;98:68‐77. 26804254 10.1016/j.freeradbiomed.2016.01.012

[67] Zhao L, Cui L, Jiang X, et al. Extracellular pH regulates autophagy via the AMPK‐ULK1 pathway in rat cardiomyocytes. FEBS Lett. 2016;590:3202‐3212. 27531309 10.1002/1873-3468.12359

[68] Genders AJ, Martin SD, McGee SL, et al. A physiological drop in pH decreases mitochondrial respiration, and HDAC and Akt signaling, in L6 myocytes. Am J Physiol Cell Physiol. 2019;316(3):C404‐C414. 30649921 10.1152/ajpcell.00214.2018

